# Comparison of radiography and ultrasonography in the detection of lung and liver cysts in cattle and buffaloes

**DOI:** 10.14202/vetworld.2016.1113-1120

**Published:** 2016-10-20

**Authors:** Ashwani Kumar, Narinder Singh Saini, Jitender Mohindroo, Balbir Bagicha Singh, Vandana Sangwan, Naresh Kumar Sood

**Affiliations:** 1 Department of Veterinary Surgery and Radiology, Guru Angad Dev Veterinary and Animal Sciences University, Ludhiana - 141 004, Punjab, India; 2 School of Public Health and Zoonoses, Guru Angad Dev Veterinary and Animal Sciences University, Ludhiana - 141 004, Punjab, India; 3 Department of Teaching Veterinary Clinical Complex, Guru Angad Dev Veterinary and Animal Sciences University, Ludhiana - 141 004, Punjab, India

**Keywords:** bovine, echinococcus cyst, liver, lung, radiography, ultrasound

## Abstract

**Aim:**

Echinococcosis is the major cause of lung and liver cysts in ruminants. This study compared usefulness of radiography and ultrasonography (USG) in the detection of lung and/or liver cysts in sick bovine animals. The study also worked out cooccurrence of lung and liver cysts, and whether these cysts were primary cause of sickness or not.

**Materials and Methods:**

This study was conducted on 45 sick bovine (37 buffaloes and 8 cattle) suffering from lung and liver cysts. A complete history of illness and clinical examination was carried out. Lateral radiographs of chest and reticular region were taken. In radiographically positive or suspected cases of cysts, USG of the lung and liver region was done. Depending on the location of cyst and clinical manifestations of the animal, the cysts were categorized as primary or secondary causes of sickness.

**Results:**

Using either imaging technique, it was observed that 46.7% of the animals had both lung and liver cysts, whereas 33.3% had only lung and 20% had only liver cyst. Cysts were identified as primary cause of sickness in 31.1% animals only. For diagnosing lung cysts, radiography (71.1%) and USG (62.2%) had similar diagnostic utility. However, for detecting liver cysts, USG was the only imaging tool.

**Conclusion:**

The lung and liver cysts, depending on their number and size may be a primary cause of sickness in bovine. Radiography and USG are recommended, in combination, as screening tools to rule out echinococcosis.

## Introduction

Echinococcosis is one of the important emerging and re-emerging parasitic zoonotic diseases affecting humans and livestock [1,2]. It is mainly caused by larvae of the tapeworm *Echinococcus granulosus* and is endemic in the Middle East countries including India [3-6]. Canids, mostly dogs, are definitive hosts, where this tapeworm grows to adulthood, in the intestines. Infection to intermediate hosts, such as ruminants and human beings, spreads through feco-oral route [1]. Bovine animals, suffering from lung and liver hydatid cysts, have been reported to remain asymptomatic, and the cystic lesions are usually diagnosed at necropsy or slaughter [2,7-11].

Non-availability of reliable tests in the diagnosis of hydatid cysts in live animals is a serious problem and necropsy is the only reliable tool. Antemortem detection of lung and liver cysts may be useful in assessing severity of the disease condition, and therefore, such animals may be culled to prevent spread of this deadly disease. Ultrasonography (USG) has been reported to detect hepatic cysts in small ruminants [12-14]. However, as per author’s knowledge, cited literature lacks comparative studies on the radiography and USG findings of lung and liver cysts in bovine.

Therefore, this study was planned to compare the usefulness of radiography and USG in the detection of lung and/or liver cysts in sick bovine animals. The study also aimed to find out cooccurrence of lung and liver cysts in cattle and buffaloes, and whether cysts were the primary cause of sickness or not.

## Materials and Methods

### Ethical approval

The approval of the Institutional Animal Ethical Committee to carry out this study was not required because this study was conducted on clinical cases presented to the Department of Veterinary Surgery and Radiology for radiographic and ultrasonographic investigation. Parameters investigated were a part of the clinical assessment of the case.

### Animals, history, and physical examination

This study included 45 sick bovine animals (37 buffaloes and eight cattle) presented at the University Veterinary Hospital and were diagnosed with cyst lesions in lungs and/or liver. These animals were suspected of forestomach, abdominal and/or lung affections. During the investigation, these animals were found positive for one or more lung and/or liver cysts. A complete history of illness and clinical examination was carried out at the time of presentation to correlate the current sickness of the animal with the diagnosis of cysts.

### Radiographic examination

**Figure-1:**
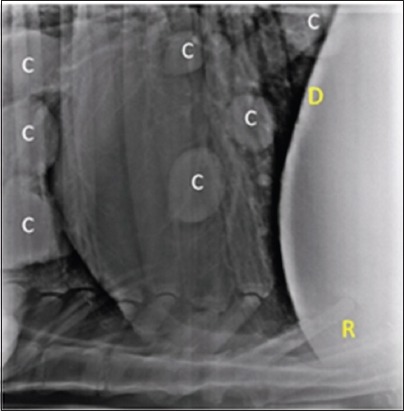
Lateral radiograph of the chest in cow showing round, discrete, single or multiples lesions of soft tissue radio-opacity in the lung region suggestive of cysts (C). Diaphragmatic line (D), reticulum (R).

Lateral radiographs of chest and reticular region were taken, using kVp 90-115, mAS 50-70, FFD 90 cm, by an 800 mA large animal X-ray machine (Siemens India, Mumbai). Radiographs were processed using computed radiography system (Kodak, India). Round to oval masses of soft tissue opacity, scattering randomly in lung region, were diagnosed as cyst lesions (Figure-1).

### Ultrasonographic examination

**Figure-2:**
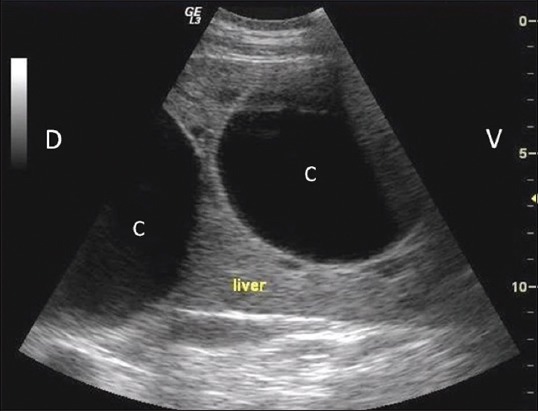
Ultrasonographic image showing two encapsulated thin walled cavitary lesions (C) in the hepatic parenchyma in buffalo. Dorsal (D), ventral (V).

**Figure-3:**
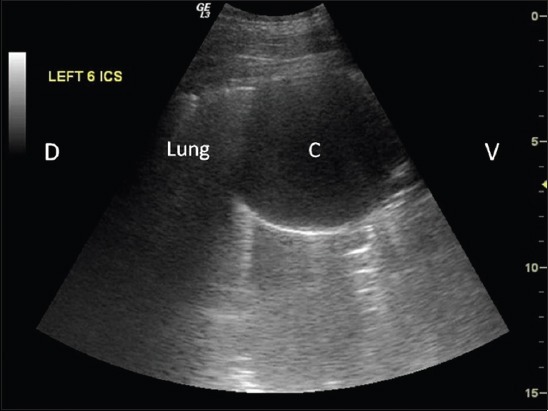
Ultrasonographic image showing cyst lesion (C) on the lung surface of buffalo. Dorsal (D), ventral (V).

**Figure-4:**
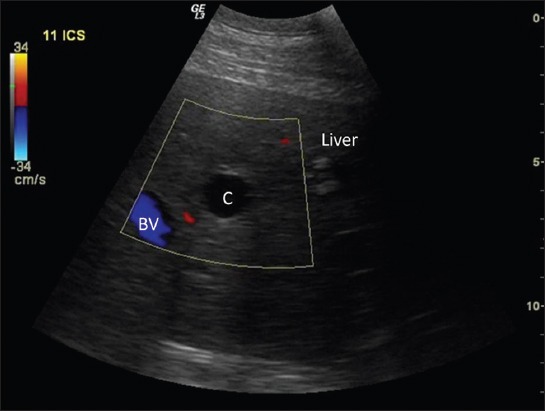
Ultrasonographic image showing color Doppler mode to confirm cavitary lesions (C) from blood vessels (BV) in the hepatic parenchyma.

For ultrasonographic scanning, animals were prepared by clipping hair from lung (both sides) and liver (right side) region. The multifrequency transducer (convex 2.0-5.0 MHz and/or linear 7.0-12.0 MHz) was moved dorsoventrally at each intercostal space in standing animals using Wipro Logiq III Expert Ultrasound Machine. Detection of any cavitary lesion in the hepatic parenchyma (Figure-2) or in lung region/surface (Figure-3) was used as a criterion in the detection of cyst. Scanning in longitudinal and transverse planes and color Doppler USG was used to confirm cavitary lesions (Figure-4). Ultrasound guided fine (22-gauge) needle aspiration of cyst lesions (n=14, 12 buffaloes, and 2 cattle) were carried out under aseptic conditions. Wet smears of aspirated fluid were examined microscopically.

### Confirmatory diagnosis

**Figure-5:**
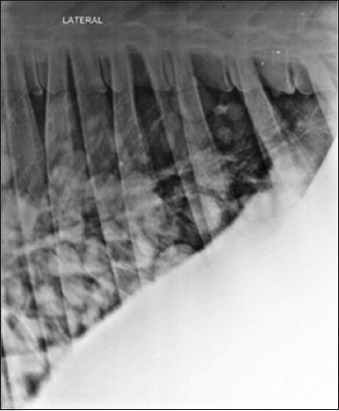
Lateral radiograph of chest showing severe diffuse nodular interstitial lung pattern suggestive of multiple cystic lesions in a cow.

**Figure-6:**
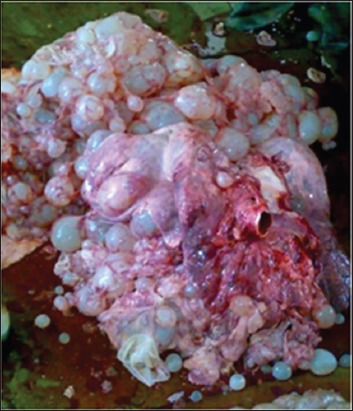
Photograph showing multiple cyst lesions in lung of cow on necropsy.

**Figure-7:**
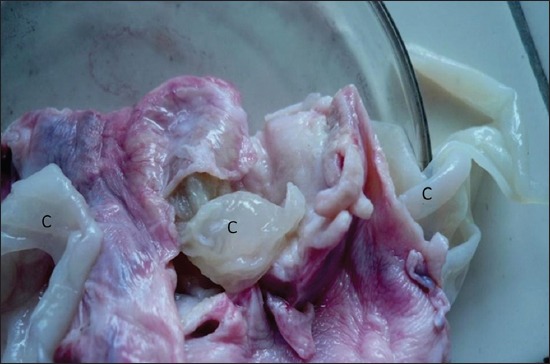
Photograph showing germinal layer of cyst (C) wall in a buffalo confirming echinococcosis.

**Figure-8:**
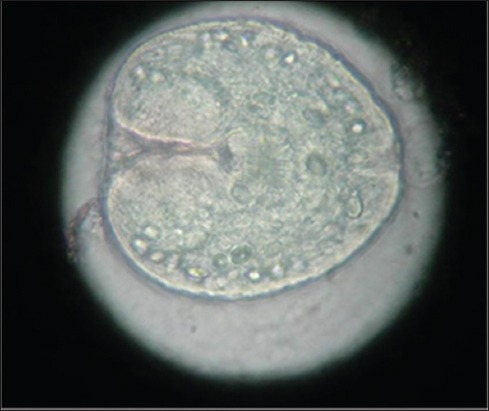
Microscopic photograph showing protoscoleces in the germinal layer of the cyst wall in a buffalo confirming echinococcosis (4×).

During the investigation, two buffaloes and one cow having extensive lung cyst lesions (Figure-5) and severe respiratory distress, died. Necropsy was done to confirm the imaging findings. Cyst fluid and germinal layer of cyst wall was examined microscopically. The final diagnosis of echinococcus cysts was made on postmortem, from the typical cystic lesions in the lung and liver parenchyma (Figures-6 and 7) and cytology of the germinal layer of cyst wall (Figure-8) in these three bovine cadavers. The diagnosis of hydatid cysts in the remaining animals was made from the typical cyst lesions in the lung and liver region on ultrasound or radiograph, needle aspiration findings and the endemic nature of the disease in Northern India.

## Results and Discussion

### Animals, history, and physical examination

This study included 37 buffaloes and 8 cattle, diagnosed with hepatic and/or lung cysts. These were adult females with a mean age of 7.15±0.35 years (range 4-15 years) and had partial (n=17; 15 buffaloes and 2 cattle) or total anorexia (n=28; 22 buffaloes and 6 cattle) since 1-4 weeks. Other clinical signs are depicted in Table-1. Similar to the observations of this study, the highest prevalence of echinococcosis was found in older animals [7,11,12]. The prevalence rate in females has been reported significantly higher as compared to males [10,13]. This could be because of the fact that females are kept for long periods due to production or reproduction reasons and are usually culled at later age [13].

**Table-1:** Clinical signs depicted by bovine suffering lung and/or liver cyst.

Clinical signs	Partial or total anorexia only	Respiratory distress	Brisket/edema	Loss of defecation	Tympany
Partial anorexia (n=17)	2	5	1	2	7
Total anorexia (n=28)	5	9	4	9	1
Total (n=45)	7	14	5	11	8

**Figure-9:**
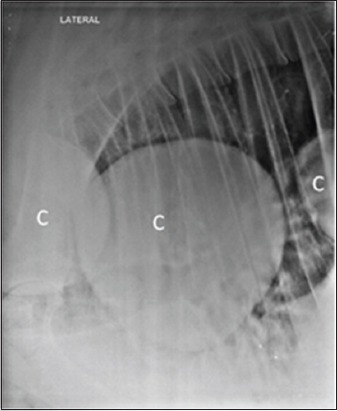
Radiograph showing massive cyst lesions (C) present near thoracic esophagus in a buffalo having persistent tympany.

**Figure-10:**
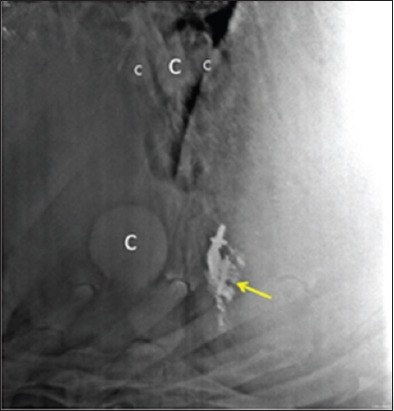
Lateral radiograph showing cystic lesion (C) of soft tissue radiopacity, in the lung region. Metallic foreign bodies (arrow) are also seen in the reticulum (R).

Cited literature reports that cystic echinococcosis in cattle is an asymptomatic disease [4]. However, clinical signs may vary and depend on the number, size, and severity of cystic lesions, resulting in impaired function of the respective organ [1,15]. Respiratory distress or open mouth breathing, in this study, could be correlated to size, location or severity of lesions in the lung parenchyma [15]. The presence of extensive cystic lesions involving the diaphragm, pleural cavity, and lungs (Figures-5 and 6) lead to reduced functional space of the lung. Moreover, this abnormality might be the cause of respiratory distress, in this study. Cyst lesions present in the region of thoracic esophagus or cardia (Figure-9) might be the cause of tympany, in this study, due to impaired eructation. However, traumatic reticulitis and/or adhesions (Figure-10) might also cause recurrent tympany in bovine animals. The majority of the animals of this study had a few small-sized cysts present in the lung or liver region which were considered non-significant to cause illness in the sick animals of this study. Moreover, in these animals, the primary cause of illness was present elsewhere. In addition to the presence of cysts, multiple associated disease conditions were diagnosed in these bovine (Table-2).

**Table-2:** Associated disease conditions in bovine having lung or liver cysts.

Disease condition	Buffalo	Cattle
Diaphragmatic hernia (n=3)	3	0
Traumatic reticuloperitonitis (n=14)	9	5
Intestinal obstruction (n=6)	5	1
Cecal dilatation (n=5)	2	3
Pericarditis (n=1)	0	1
Grand total (n=29)	19	10

However, one buffalo which was presented with no apparent respiratory abnormality but developed severe respiratory distress only when it was casted on the ground for radiography and later died within a few minutes. Radiographic and postmortem examination of this buffalo confirmed multiple giant hydatid cysts in the lungs.

### Radiographic findings

Lateral radiographs of the chest revealed various radiographic findings in bovine having lung or liver cysts as depicted in Table-3. Radiography of the liver, in bovine animals, was not possible to detect liver cysts. Radiography has been reported to be a reliable tool in diagnosing lung pathology [16,17]. In this study, round to oval masses of soft tissue opacity, scattering randomly in the lung parenchyma (Figure-1), were observed in the majority of the cases, suffering from cysts. Moreover, this conforms to the previous findings, reported in cattle [18] and human beings [1,15].

**Table-3:** Radiographic findings in bovine having lung cysts.

Radiographic signs	Buffalo	Cattle	Total
Single or multiple soft tissue opacities in lungs	25	7	32
Fluid opacity in ventral lungs	4	0	4
Marked nodular interstitial lung pattern	3	1	4
No apparent abnormality	5	0	5
Total	37	8	45

### Ultrasonographic findings

USG was sufficient to detect single or multiple, encapsulated, thin or thick walled cavitary lesions, with anechoic to hyperechoic contents, within the hepatic parenchyma of 68.9% animals (n=31; 24 buffaloes and 7 cattle) (Figure-2). Scanning in longitudinal and transverse planes and color Doppler USG (Figure-4) was useful to confirm cavitary lesions. In the remaining 14 animals (13 buffaloes and one cow), no cyst lesion was detected in the hepatic parenchyma; however, radiographically cyst lesions were apparent in the lung region.

**Figure-11:**
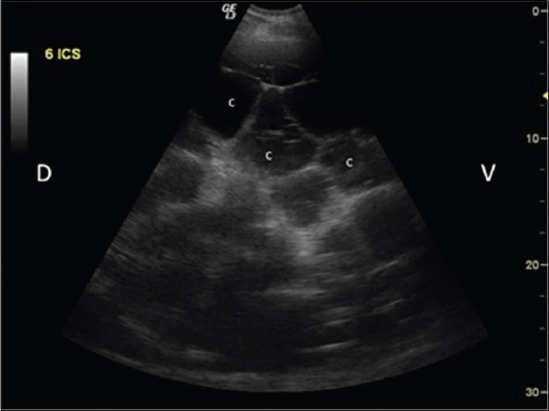
Ultrasonographic image showing multiple cysts (C) extending on to the diaphragm, pleural cavity, and lungs in a buffalo. Dorsal (D), ventral (V).

Of 71.1% (32/45) cases showing round to oval radiopaque lesions on radiographs (Figures-1, 5, 9, and 10), in 75% animals (24/32) lung cysts could be detected using thoracic ultrasound (Figures-3 and 11). Apart from this, four more cases were positive for lung cysts on ultrasound which were not appreciable on radiography. Hence, a total of 36 bovine were positive for lung cysts using radiography or USG technique. Out of these 36 cases of lung cysts, 22 animals (58.5%) also had liver cysts on USG suggestive of high co-occurrence of lung and liver cysts in bovine. In the lung region, cysts were detected either as single or multiple; whereas in the liver, majority of the animals had multiple cysts, as depicted in Table-4.

**Table-4:** Detection of single or multiple lung or liver cyst using USG and/or radiography.

Number of cysts	Single cysts		Total	Multiple cysts		Total	Grand total
	
	Buffalo	Cattle		Buffalo	Cattle		
Lung (n=36)	14	1	15	15	6	21	36
Liver (n=31)	2	0	2	22	7	29	31

USG: Ultrasonography

**Figure-12:**
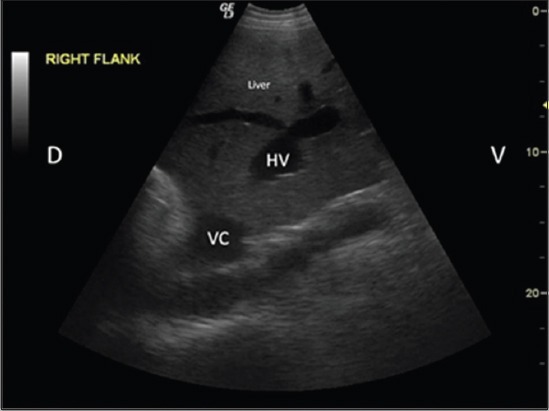
Ultrasonographic image of liver showing round vena cava (VC) and dilated hepatic veins (HV). Dorsal (D), ventral (V).

Of 21 animals (15 buffaloes and six cattle) suffering from multiple cystic lesions, in five buffaloes and three cattle, the cystic lesions were extensive and extending onto the diaphragm, pleural cavity, and lungs (Figures-5 and 11) and were also present in the liver. Of these, two buffaloes and one cow died and necropsy (Figure-6) confirmed the radiographic and ultrasonographic findings. USG of four buffaloes and one cow having brisket edema also showed marked hepatomegaly characterized by round liver margins and scanning of the liver at the right flank and ventral to the imaginary line between the right hip bones to elbow as described previously [12]. Hepatic congestion was characterized by dilated and persistently round posterior vena cava (Figure-12). Hepatic congestion and hepatomegaly was also present in one cow having single lung cyst and pericarditis. Marked hepatomegaly, in this study, could be correlated to the presence of space occupying lesions in the hepatic parenchyma or hepatic congestion. Hepatic dysfunction might be the cause of anorexia, in this study. Persistent dilatation of caudal vena cava is indicative of systemic congestion. It may be caused by cardiac insufficiency due to pericarditis, thrombosis of the caudal vena cava and compression of the caudal vena cava in the thorax or in the subphrenic region by space-occupying lesions [19-21]. Cyst lesions present near diaphragm or right atrium might have caused pressure over right atrium or posterior vena cava leading to dilatation of vena cava and systemic congestion, in this study.

### Comparison of radiography and USG

Using both imaging techniques, cysts were detected either in both lung and liver (46.7%; 15 buffalo and 6 cattle) or only in the lung (33.3%; 14 buffalo and 1 cow) or liver (20.0%; 8 buffalo and 1 cow). Radiography alone diagnosed cysts in 71.1% animals (32/45) present in lung region while ultrasound alone diagnosed lung cysts in 62.2% (28/45) animals and liver cysts in 68.9% (31/45) animals. Radiography was found more sensitive than USG for diagnosing lung cysts. However, USG was the only imaging modality to detect liver cysts. Both imaging techniques were found complementary to each other in the detection of lung and liver cysts, therefore, combined use of radiography and USG can increase the efficacy of diagnosing cysts.

Out of 71.1% (32/45) cases showing round to oval radiopaque lesions on radiographs (Figure-5), in 75% animals (24/32) lung cysts could be detected using thoracic ultrasound (Figure-6). Out of total 36 cases diagnosed with lung cysts, using either imaging technique, 21 animals (58.3%) also had liver cysts on USG. Cyst lesions were present either as single (33.3%; 14 buffaloes and one cow) or multiple (46.7%; 15 buffaloes and six cattle) in the lung region. However, in the liver region, 64.5% animals (22 buffaloes and seven cattle) had multiple cysts whereas in only 2 buffaloes (4.4%) had a single liver cyst (Table-5).

**Table-5:** Diagnosis of lung or liver cysts based on USG or radiography in bovine.

Radiography		USG		Both radiography and USG		Total
			
Buffalo	Cattle	Buffalo	Cattle	Buffalo	Cattle	
14	1	8	1	15	6	45

USG: Ultrasonography

**Figure-13:**
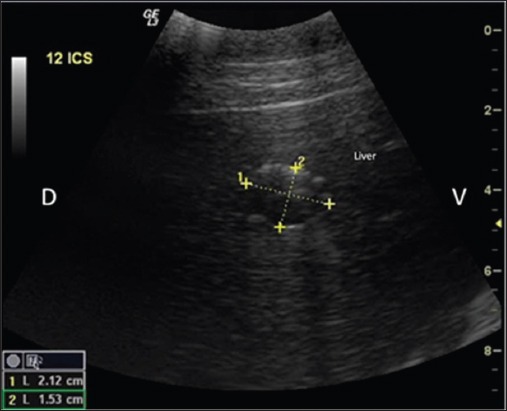
Ultrasonographic image showing cyst lesions with thickened wall in liver. Dorsal (D), ventral (V).

USG was found useful in detecting hepatic cysts present within hepatic parenchyma (Figures-2 and 13), and lung cysts located close to the visceral pleura (Figure-3). However, it was not possible to detect lung cysts lying deep within the lung parenchyma with USG which were otherwise detected on radiography, as the aerated lungs act as barrier to ultrasound [12,22-24]. Similarly, it is reported that lung abscesses/lesions located either close to visceral pleura or extending up to the pleura can only be diagnosed on ultrasound [16,17,25,26]. Radiography has been reported to diagnose lung cysts in cattle [18] but comparative study on radiography and USG for diagnosing lung and liver cysts has not been cited in the literature.

Slaughterhouse-based studies revealed that hydatid cysts may be found either in the lungs or in the liver or in both the liver and lungs of ruminants [7,9,11-13,15,22,23]. Cysts are rarely found in other organs such as spleen, heart, kidney, and omentum [10,27]. Lungs and liver being the major predilection sites for echinococcus cysts and thorough examination of these organs can rule out this condition, especially in endemic areas.

### Cytological findings

**Figure-14:**
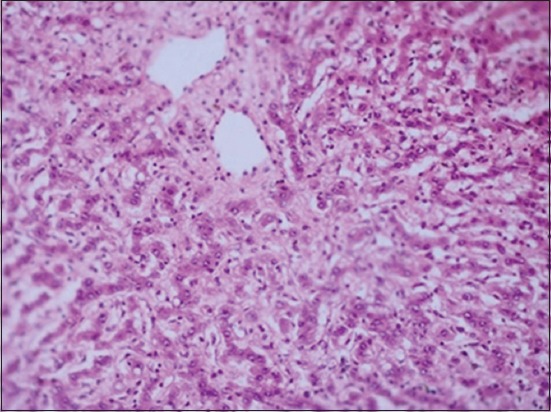
Microscopic image of liver showing pressure atrophy of hepatic cords (H and E, 10×).

Cytology of ultrasound guided cyst fluid (n=14) indicated transudate or cystic fluid, with no evidence of parasites, indicating sterile nature of hepatic/lung cysts. However, microscopic examination of the germinal layer of cyst wall demonstrated Protoscoleces in three bovine cadavers, confirming echinococcosis (Figures-7 and 8). Histology of liver biopsy adjoining cysts (Figure-2) revealed pressure atrophy of the hepatic cords (Figure-14).

**Figure-15:**
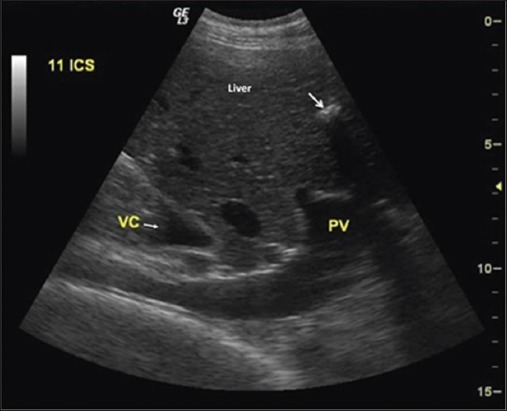
Ultrasonographic image of liver showing calcified cyst lesion (arrow) with prominent acoustic shadow. Portal vein (PV), vena cavae (VC).

Echinococcus cysts may be either sterile or fertile, depending on their stage of development. Cysts in the initial stages of development may be sterile and become fertile and later degenerate in due course of time. In this study, the majority of the cysts were purely cystic suggesting initial stages of disease. Moreover, these cysts were sterile, because of their initial stage, as revealed by cytology. Cysts with wavy, irregular wall or calcified margins (Figure-15) correspond to degenerating or dead cysts [28,29] or calcified hepatic abscesses [30]. Microscopic examination of germinal layer of cyst wall is generally diagnostic based on visualization of protoscoleces [1,5,6]. Being clinical study, it was not possible to identify the exact etiology of the cysts in all the cases of this study. However, the high regional prevalence of echinococcosis and detection of typical cyst lesions in lung and liver were suggestive of hydatid cysts. Moreover, cysts lesions were identified as echinococcus cysts in three bovine cadavers, in this study.

Various causes of cavitary lesions within the hepatic parenchyma are abscess, neoplasia or dilated bile ducts and cysts [31]. These cavitary lesions can be distinguished based on macroscopic nature of the needle aspirated contents and cytological findings [31]. Needle aspiration of cavitary lesions is recommended under ultrasound guidance. It facilitates correct placement of needle into the lesion and prevents accidental puncture of major blood vessel.

In this study, the majority of the animals had both liver and lung cysts. As USG could detect both lung and liver cysts, it is suggested that USG would be used as a potential mass screening tool in the detection of cystic echinococcosis in cattle and buffaloes. General practitioners, with no previous exposure of USG, may be easily trained to carry out autonomous preliminary ultrasound surveys in echinococcus endemic areas [32]. They can assist to study the prevalence, monitoring, efficacy of treatment, and follow-up of echinococcus cases in remote areas, having no accessibility of sonologist [32]. High predictive value of ultrasound for the diagnosis of hydatidosis in sheep and goat has been reported previously [12,13,22-24]. USG has also been stated to diagnose hepatic cysts in human beings [28,29,32-34] and cattle [35]. Published literature on the application of USG in the detection of lung and liver cysts in buffaloes is not available. Moreover, it is reported that seroepidemiological surveys for the diagnosis of cystic echinococcosis require better diagnostic antigens and its findings should be supported by imaging methods such as USG which is quick and relatively less expensive than other imaging techniques [32-34]. In addition, USG facilitates needle aspiration of cyst contents that may confirm and differentiate various hepatic cavitary lesions such as abscess, dilated bile duct, carcinoma based on gross appearance, cytology, and bacteriology [31].

### Cysts being primary or secondary cause of illness

In 16 animals (13 buffaloes and 3 cattle), cysts were identified as primary cause of sickness which consisted of multiple lung cysts (n=8, 5 buffaloes and 3 cattle), multiple/giant liver cysts leading to marked echotextural changes in hepatic parenchyma (Figure-2) of 3 buffaloes and persistent tympany due to giant lung cysts in the region of thoracic esophagus (Figure-9) in 5 buffaloes. One clinical study reported giant hydatid lung cyst as a cause of respiratory discomfort in pediatric patients [15].

In this study, bovine animals diagnosed with lung and/or liver cysts, also had one or more significant pathological lesions such as reticular diaphragmatic hernia in buffaloes, linear metallic foreign bodies in reticulum (Figure-10), cecal dilatation, intestinal obstruction, and pericarditis (Table-2) suggesting cysts being secondary cause of sickness. Similar findings have been reported by various authors [2,7-11]. However, depending on the location and/or size of the cystic lesions, these may be diagnosed as primary causes of sickness in bovine animals.

Echinococcosis is a disease of considerable zoonotic importance. Dogs being definitive hosts for echinococcosis, keeping infected dogs along with farm animals can transmit infection. Eating infected flesh of bovine cadavers by the dog completes the life cycle of the parasite. Regular deworming of domestic dogs against tapeworm and proper disposal of infected carcass of ruminants has been recommended to prevent the spread of echinococcosis [1].

## Conclusions

The following conclusions were drawn from this study:

1. Lung and liver cysts, depending on their number and size may be a primary cause of sickness in bovine
2. USG is a simple and safe technique for the detection of lung cysts extending up to the visceral pleura
3. USG is an exclusive imaging technique to diagnose liver cysts in bovine
4. Combined use of radiography and USG is recommended as screening tools to detect lung and liver cysts in cattle and buffaloes in endemic areas.

## Authors’ Contributions

AK, NSS, and JM designed this clinical study. AK, JM, VS, NSS performed radiological and ultrasonographic interpretations. NKS conducted necropsy and histopathological examination. BBS performed cytological examination of cyst fluid and germinal layer of cysts to confirm the diagnosis. All the authors read, revised, and approved the final manuscript.
